# An Epidemiological Analysis of SARS-CoV-2 Genomic Sequences from Different Regions of India

**DOI:** 10.3390/v13050925

**Published:** 2021-05-17

**Authors:** Pragya D. Yadav, Dimpal A. Nyayanit, Triparna Majumdar, Savita Patil, Harmanmeet Kaur, Nivedita Gupta, Anita M. Shete, Priyanka Pandit, Abhinendra Kumar, Neeraj Aggarwal, Jitendra Narayan, Neetu Vijay, Usha Kalawat, Attayur P. Sugunan, Ashok Munivenkatappa, Tara Sharma, Sulochna Devi, Tapan Majumdar, Subhash Jaryal, Rupinder Bakshi, Yash Joshi, Rima Sahay, Jayanti Shastri, Mini Singh, Manoj Kumar, Vinita Rawat, Shanta Dutta, Sarita Yadav, Kaveri Krishnasamy, Sharmila Raut, Debasis Biswas, Biswajyoti Borkakoty, Santwana Verma, Sudha Rani, Hirawati Deval, Disha Patel, Jyotirmayee Turuk, Bharti Malhotra, Bashir Fomda, Vijaylakshmi Nag, Amita Jain, Anudita Bhargava, Varsha Potdar, Sarah Cherian, Priya Abraham, Anjani Gopal, Samiran Panda, Balram Bhargava

**Affiliations:** 1 Indian Council of Medical Research-National Institute of Virology (ICMR-NIV), Pune 411021, India; hellopragya22@gmail.com (P.D.Y.); nyayanit.dimpal@gmail.com (D.A.N.); triparna.majumdar@gmail.com (T.M.); varshapatil111@yahoo.com (S.P.); anitaaich2008@gmail.com (A.M.S.); priyanka.pb83@gmail.com (P.P.); abhinendra.biotech@gmail.com (A.K.); yashjos1401@gmail.com (Y.J.); dr.rima.sahay@gmail.com (R.S.); potdar.v@gov.in (V.P.); sarahcherian100@gmail.com (S.C.); director.niv@icmr.gov.in (P.A.); 2 Indian Council of Medical Research, New Delhi 110029, India; harmanmeet.kaur@gmail.com (H.K.); aggarwal.n@icmr.gov.in (N.A.); jitunarayan@gmail.com (J.N.); drneetuvijay@gmail.com (N.V.); pandasamiran@gmail.com (S.P.); balram.bhargava@gov.in (B.B.); 3 Sri Venkateswara Institute of Medical Sciences, Tirupati 517507, India; ukalawat@yahoo.com; 4 ICMR—NIV Field Unit, Kerala 688005, India; apsugunan@gmail.com; 5 ICMR-NIV, Bangalore Unit, Bangalore 560029, India; ashokmphdns@gmail.com; 6 VRDL Sikkim Government College of Nursing, Gangtok 737101, India; stnmvdl17@gmail.com; 7 Regional Institute of Medical Sciences IMPHAL, Imphal 795004, India; sulo_khu@rediffmail.com; 8 Government Medical College, Agartala, Tripura 799006, India; drtapan1@rediffmail.com; 9 Dr. Rajendra Prasad Government Medical College, (H.P.), Kangra 176001, India; drscjaryal@gmail.com; 10 Government Medical College, Patiala 147001, India; rupindergill1@yahoo.co.in; 11 Kasturba Hospital for Infectious Diseases, Mumbai 400034, India; jsshastri@gmail.com; 12 Post Graduate Institute of Medical Education & Research, Chandigarh 160012, India; minipsingh@gmail.com; 13 Rajendra Institute of Medical Sciences, Ranchi 834009, India; manoj_drmicro@rediffmail.com; 14 Government Medical College, Haldwani 263129, India; drvinitarawat@gmail.com; 15 National Institute of Cholera and Enteric Diseases, Kolkata 700010, India; shanta1232001@yahoo.co.in; 16 Bhagat Phool Singh Government Medical College, Sonipat 131305, India; yadav78sarita@yahoo.com; 17 King Institute of Preventive Medicine & Research, Chennai 600032, India; kaveri_raj1967@yahoo.com; 18 Indira Gandhi Government Medical College & Hospital, Nagpur 440018, India; sharmilakuber@gmail.com; 19 All India Institute of Medical Sciences, Bhopal 462020, India; debasis.microbiology@aiimsbhopal.edu.in; 20 Regional Medical Research Centre, Dibrugarh 786010, India; biswaborkakoty@gmail.com; 21 Indira Gandhi Medical College & Hospital, Shimla 171001, India; santwana1812@gmail.com; 22 Osmania Medical College, Hyderabad 500095, India; sudhavannavada1965@gmail.com; 23 Regional Medical Research Center Gorakhpur, Gorakhpur 273013, India; dr.hirawati@gmail.com; 24 B.J. Medical College, Ahmedabad 380016, India; poliobjmedical@gmail.com; 25 RMRC, Bhubaneswar 751023, India; drjyotirmayeeturuk@gmail.com; 26 Sawai Man Singh Medical College, Jaipur 302004, India; drbhartimalhotra@gmail.com; 27 Sher-I-Kashmir Institute of Medical Sciences, Srinagar 190011, India; bashirfomda@gmail.com; 28 All India Institute of Medical Sciences, Jodhpur 342005, India; vijayalakshmi005@gmail.com; 29 Department of Microbiology, King George’s Medical University, Lucknow 226003, India; amita602002@yahoo.com; 30 All India Institute of Medical Sciences, Raipur, Raipur 492099, India; anuditabhargava@gmail.com; 31 Indian Institute of Science Education and Research, Pune 411008, India; anjirao95@yhoo.com

**Keywords:** SARS-CoV-2, epidemiology, NGS, clades, India

## Abstract

The number of Severe Acute Respiratory Syndrome Coronavirus-2 (SARS-CoV-2) cases is increasing in India. This study looks upon the geographic distribution of the virus clades and variants circulating in different parts of India between January and August 2020. The NPS/OPS from representative positive cases from different states and union territories in India were collected every month through the VRDLs in the country and analyzed using next-generation sequencing. Epidemiological analysis of the 689 SARS-CoV-2 clinical samples revealed GH and GR to be the predominant clades circulating in different states in India. The northern part of India largely reported the ‘GH’ clade, whereas the southern part reported the ‘GR’, with a few exceptions. These sequences also revealed the presence of single independent mutations—E484Q and N440K—from Maharashtra (first observed in March 2020) and Southern Indian States (first observed in May 2020), respectively. Furthermore, this study indicates that the SARS-CoV-2 variant (VOC, VUI, variant of high consequence and double mutant) was not observed during the early phase of virus transmission (January–August). This increased number of variations observed within a short timeframe across the globe suggests virus evolution, which can be a step towards enhanced host adaptation.

## 1. Introduction

The severe acute respiratory syndrome coronavirus-2 (SARS-CoV-2) that was first identified in Wuhan City in the Hubei Province of China in December 2019 has now spread worldwide [1]. The causative agent was identified as SARS-COV-2 based on laboratory diagnoses and the study of its genome through next-generation sequencing (NGS) [2]. SARS-CoV-2 belongs to the subgenus *Sarbecovirus* of the *Betacoronavirus* genus that is part of the *Coronaviridae* family, which has one of the largest RNA viral genomes. It is an enveloped, non-segmented, positive-sense RNA virus, with a genome approximately 30 kb in length [3]. The first ten whole-genome sequences of SARS-CoV-2 obtained from nine affected patients in China showed 99.98% sequence identity with each other, and ~96.3% identity with a bat Coronavirus (BatCoV) strain, RaTG13 [4].

A wide range of studies has been published to describe the geographic distribution of SARS-CoV-2 clades and variants across the world using whole-genome sequencing (WGS). WGS has emerged as an important tool for understanding the effect of mutations on disease transmission dynamics, and for predicting the trends of the ongoing pandemic. Several studies have reported genetic variations in the virus resulting from different types of mutations: missense, synonymous, insertion, deletion, and non-coding mutations [5,6,7]. Recently, the Center for Disease Control (CDC) classified SARS-CoV-2 variants into three groups: “variant of interest (VUI)”, “variant of concern”, and “variant of high consequence” (https://www.cdc.gov/coronavirus/2019-ncov/cases-updates/variant-surveillance/variant-info.html (accessed on 26 April 2021)). The B.1.526, B.1.525, P.2 Phylogenetic Assignment of Named Global Outbreak LINeages (PangoLIN) lineages are classified as VUIs, whereas the B.1.1.7, B.1.351, B.1.427, B.1.429, P.1 PangoLIN lineages are classified as VOCs.

The present study was designed to detect the genetic diversity of SARS-CoV-2 strains sampled from different states in India, which were taken monthly at different time points and which demonstrated the presence of mutations in the SARS-CoV-2 variant as described by the CDC, among other institutions. This study reveals the circulating lineages of SARS-CoV-2 in India and also highlights the mutational rates of the virus in different geographic regions in India over a consistent period of six months (January–August 2020).

## 2. Materials and Methods

### 2.1. Sample Acquisition

Respiratory specimens i.e., nasopharyngeal/oropharyngeal swabs (NPS/OPS) positive for SARS-CoV-2 detected by real-time RT-PCR, were collected monthly (from 1 January to 31 August 2020) from Virus Research and Diagnostic Laboratories (VRDLs) throughout India and sequenced. Samples were collected from 25 states and Union Territories (UTs) in India (Andhra Pradesh, Assam, Chandigarh, Chhattisgarh, Delhi, Gujarat, Haryana, Himachal Pradesh, Jammu and Kashmir, Jharkhand, Karnataka, Kerala, Madhya Pradesh, Maharashtra, Manipur, Odisha, Punjab, Rajasthan, Sikkim, Tamil Nadu, Tripura, Telangana, Uttarakhand, Uttar Pradesh, and West Bengal). The inclusion criteria for the samples in the study were the following: (i) appropriate storage of the sample at −80 °C, and (ii) a cycle threshold of <30. The samples fulfilling the above criteria were packed in dry ice and in triple-layer packaging, according to guidelines established by the International Air Transport Association (IATA). The samples were subsequently transferred to the nodal laboratory—the Indian Council of Medical Research-National Institute of Virology (ICMR-NIV) in Pune—for sequencing and analysis. Apart from the sequences of the above-collected samples, all other sequences of the SARS-CoV-2 collected in India from January to August and deposited in the Global Initiative on Sharing All Influenza Data (GISAID) by various researchers, updated until 9 December 2020, were also analyzed.

### 2.2. RNA Extraction and Next-Generation Sequencing

Viral nucleic acid was extracted from the NPS/OPS specimens using 400 μL of the sample. Nucleic acid extraction was conducted using a MagMAX™ viral pathogen nucleic acid isolation kit (Thermo Fisher Scientific, Waltham, MA, USA). An automated protocol using the KingFisher Flex (Thermo Fisher Scientific, Waltham, MA, USA) Magnetic Particle Processor for high-throughput nucleic acid extraction was used, following the instructions of the manufacturer. Nucleic acid was eluted with 50 μL of elution buffer. The extracted RNA was quantified using the Qubit^®^ 2.0 Fluorometer (Thermo Fisher Scientific, Waltham, MA, USA) with the Qubit RNA High Sensitivity kit. Host ribosomal RNA (rRNA) depletion was carried out using the NEBNext rRNA depletion kit (New England Biolabs, Ipswich, MA, USA) and the extracted RNA was re-quantified. The quantified RNA was used to generate genomic libraries for sequencing. In brief, the library preparation involved fragmentation, adapter ligation, amplification, and quantification. The quantified libraries were normalized and loaded onto the Illumina machine for sequencing [8,9].

The paired-end FASTQ files generated from the MiniSeq machine were analyzed on the CLC Genomics Workbench version 20 (CLC, Qiagen, Hilden, Germany). A reference-based assembly method, as implemented in the Workbench, was used to retrieve the SARS-CoV-2 sequence. The SARS-CoV-2 isolate Wuhan-Hu-1 (Accession No.: NC_045512.2) was used as the reference for mapping. The retrieved sequences were deposited in the public repository, GISAID.

### 2.3. Phylogenetic and Sequence Analysis

SARS-CoV-2 sequences from India (as of 9 December 2020), were downloaded (*n* = 3119) from the GISAID database, including the sequences of COVID-19 cases that occurred from January to August 2020 [10]. Representative sequences based on data from the different States/UT and the specific clades were used in the analysis along with the sequences retrieved in this study. The sequences were aligned using the CLC genomics workbench. The aligned file was manually checked for correctness. A neighbor-joining (NJ) phylogenetic tree was constructed from the coding region of the SARS-CoV-2 genome using the maximum composite likelihood model along with gamma distribution as the rate variation parameter. A bootstrap replication of 1000 cycles was performed to assess the statistical robustness of the generated tree. The amino acid variation for each gene, as well as the net nucleotide and amino acid divergence were identified using the MEGA software version 7.0 [11] and illustrated using the GraphPad Prism v9. PopART v1.7 was used to draw haplotype networks using the median-joining approach [12,13].

## 3. Results

### 3.1. Site Selection and Clinical Analysis

Altogether, 1603 samples were received from twenty-five states and UTs, 79 of which were discarded in compliance with the inclusion criteria. The data set of 1524 samples was used for the clinical and NGS analyses.

The clinical data were analyzed for the samples that gave complete SARS-CoV-2 sequences (*n* = 689). The mean age of patients in the study was 37 years, with a median of 35 years (range 25–45 years). A total of 71.45% of the samples were from male patients with a mean age of 37 years, while the remaining samples were from females with a mean of 38 years. The data set for age and gender were not available for three samples.

The clinical symptoms observed in the patients included in the study indicated that 67.95% of the males and 68.39% of the females sampled were asymptomatic. Overall, 67.34% of cases were asymptomatic, while 23.80% were symptomatic with one or more presenting symptoms (Supplementary Table S1). Symptom history was not available for the remaining 8.85% of patients. Low-grade fever was the most consistent clinical presentation, observed in 64.02% of symptomatic cases, followed by cough (51.22%), sore throat (25%), breathlessness (20.73%), headache (17.07%), body ache (11.59%), and feeling cold (9.15%). Loss of smell and taste was observed in 9.76% of cases, while nausea and vomiting were observed in 2.44% of cases. Hemoptysis was reported in a single case.

### 3.2. NGS Data Selection and Phylogenetic Analysis

**Figure 1:**
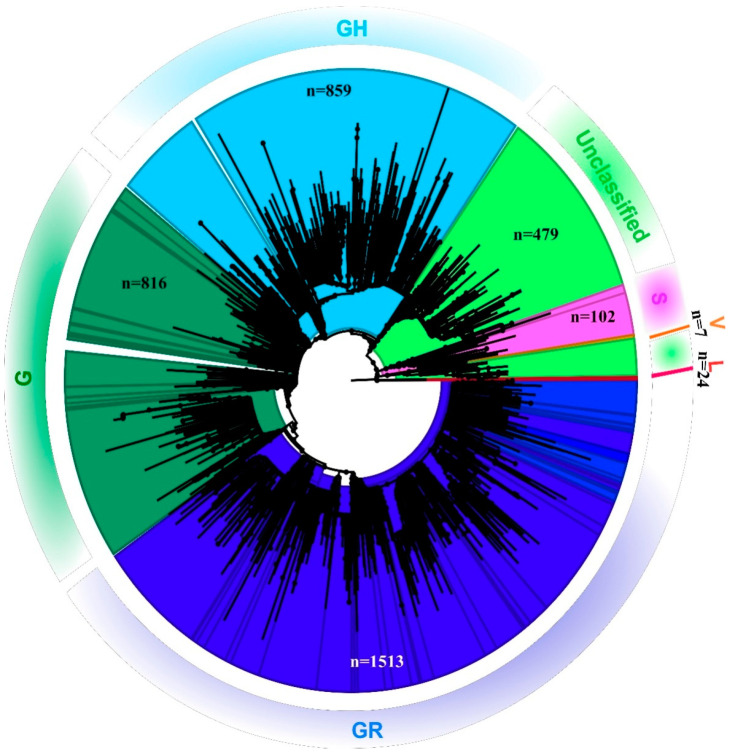
Phylogenetic tree of the SARS-CoV-2 genome: Neighbor-joining tree for Indian SARS-CoV-2 sequences with a bootstrap replication of 1000 cycles. FigTree v1.4.4 was used to visualize the generated tree. Various GISIAD clades of the SARS-CoV-2 are marked in different colors. The number of sequences used to generate the tree is assigned the *n* value.

Out of the 1524 total samples sequenced, reference-based mapping led to the retrieval of 819 SARS-CoV-2 genomic sequences that had the genome coverage of 98%. However, the study analyzed 689 complete SARS-CoV-2 genomic sequences that were retrieved for this study along with seven additional sequences. These 689 sequences had a genome coverage of more than 99.75%. A total of 696 SARS-CoV-2 sequences were analyzed in this study. The percentage of relevant mapped reads had a median value of 10.21, with an intra-quartile range of 48.29 [1.58–49.44]. Details with respect to the percentage of the genome retrieved and the read mapped to it, along with the percentage of genome length taken from each SARS-CoV-2 sequence in this study are provided in the Supplementary Table S2. The phylogenetic tree revealed that the SARS-CoV-2 sequences collected from the different states in India represent different GISAID clades with a small proportion of L and V clades [14] (Figure 1).

### 3.3. Mutations Observed in the SARS-CoV-2 Sequences

**Figure 2:**
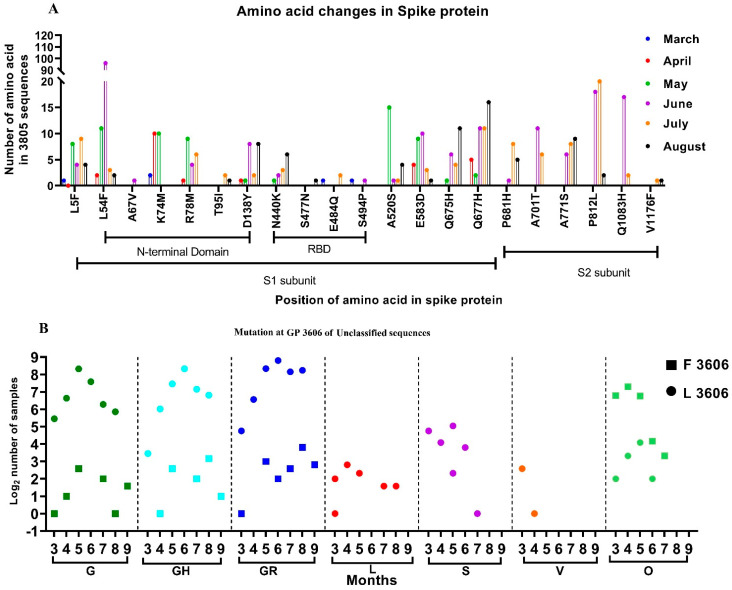
Month-wise changes in the mutations observed in SARS-CoV-2 sequences: (**A**) Month-wise changes in the amino acid mutations in the spike protein that were observed in more than 0.5% of the samples studied. (**B**) Month-wise changes in the Leucine and Phenylalanine amino acids at GP 3606, position 3606, observed within different clades.

From among the 3815 sequences analyzed, which include sequences from the GISAID database, 2473 independent amino acid variations were observed with respect to the SARS-CoV-2 isolate Wuhan-Hu-1 (Accession No.: NC_045512.2). Many individual variations observed in the SARS-CoV-2 sequences in the study were not reported. Additionally, we looked for the presence of the amino acid mutation that was reportedly found in the SARS-CoV-2 variants. However, independent amino acid mutations found in the SARS-CoV-2 variants were observed in the sequences retrieved between January and August. Figure 2A depicts the month-wise amino acid mutations observed in more than 0.5% of the sequences, along with those seen in the SARS-CoV-2 variants. It was noted that the majority of the changes occurred in the N terminal and the RBD domains of the S1 subunit of the spike protein.

Another interesting finding that warrants attention is the presence of a combination of amino acid mutations, used by the GISAID to differentiate clades. Firstly, the GR-GH variants defined by the presence of the D614G, G204R, and Q57H mutations in the S, N, and ORF3a proteins, respectively, were observed in 14 sequences from different parts of India, starting from May 2020 (Figure 2). Ten of these were from Maharashtra (*n* = 6) and Gujarat (*n* = 4), and one each from Odisha, Kerala, Chhattisgarh, and Punjab. Secondly, the GV-GR variant defined by the presence of A222V in the S and G204R mutations in the N protein, along with the common D614G mutation of the G clade, were observed starting from August 2020 in Maharashtra (*n* = 1) and Telangana (*n* = 2). Finally, the G-S variant, defined by the presence of the D614G and L84S mutations in the S and ORF8 proteins, respectively, was observed in four sequences of SARS-CoV-2 reported from Gujarat in June 2020.

The overall percentage of net evolutionary nucleotide and amino acid divergence between different clades is provided in Table 1. The lower left matrix and the upper right matrix show the percentage of nucleotide and amino acid divergence of the different clades. It can be observed that the nucleotide divergence of the clades circulating in the early phase of the pandemic is less, indicating closer evolutionary relatedness. The G clade sequences have higher divergence, which increased with the evolution of the G clade sequences into its subclades. The highest divergence from the L clade sequences was observed in the recently identified GV-GR clade sequences. However, the G-S clade sequences were found to be more similar (99.959%) to the S clade sequences rather than to the G clade sequences.

Further conservation of the missense mutation (Leucine (Leu)-Phenylalanine (Phe)) was observed at the genomic position (GP) 3606, located in the Non-Structural Protein 6 (NSP-6) of the SARS-CoV-2. We investigated this missense mutation month-wise and observed that majority of the G clade and its subclades sequences had Leucine at GP: 3606 (Figure 2B, marked as a circle). Furthermore, the SARS-CoV-2 sequences that were reported in the early pandemic (L, S, V) likewise had Leucine at this position, although with a few exceptions.

Interestingly, 12.7% of SARS-CoV-2 sequences (*n* = 3815) analyzed in this study had Phenylalanine (Figure 2B, marked as a square). The majority of these sequences (84%) are grouped in the unclassified cluster, as opposed to the sequences containing Leucine at GP: 3606. The rest of the sequences belonged to different GISIAD clades. The two sequences reported from India during January 2020, had Leucine at GP: 3606. The SARS-CoV-2 sequences deposited from India from March onwards had the Phenylalanine amino acid at GP: 3606. Figure 2 depicts the different GISAID clades that were observed month-wise and had the GP: 3606 missense mutation. This study looks upon the conservation of amino acids in the 406 unclassified SARS-CoV-2 sequences that contain Phenylalanine amino acid at GP: 3606.

**Figure 3:**
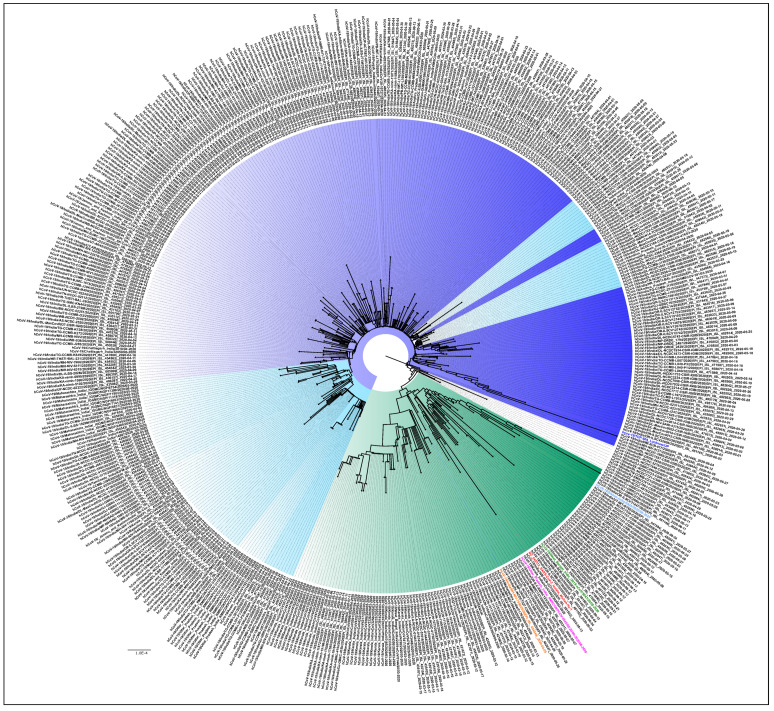
Phylogenetic tree of the 406 unclassified SARS-CoV-2 genomes: A Neighbor-joining tree of the 406 unclassified SARS-CoV-2 sequences retrieved in this study, along with the representative SARS-Cov-2 sequences from different clades with a bootstrap replication of 1000 cycles. Two major groups of unclassified sequences were observed, which are marked in different shades of blue and green. The first cluster has amino acid changes at ORF1ab (T2016K, A4489V) and N: P13L, represented in blue and its shades, whereas the second cluster has amino acid changes at ORF1ab (R207C, V378I, and M2790I), represented with the green color edges. The representative L, S, V, G, GH, GR GISAID clades are marked on the nodes with the colors red, pink, orange, green, blue, and light blue, respectively. FigTree v1.4.4 was used to visualize the generated tree.

Figure 3 is the NJ tree of the 406 unclassified sequences with the L3606F mutation, along with the representative clade sequences, which show two different clusters. In the first cluster, we can see the branches with nodes in blue and its shades, while in the second, the branches in the color green can be observed. An analysis of the unclassified sequences was performed to identify the presence of any conserved amino acid mutational pattern (*n* = 406) that led to the observed clustering. It was noted that 331/406 sequences had a conserved pattern in the ORF1ab (T2016K, A4489V) and N: P13L, Figure 3 (blue and its shades). This is identified as the B.6 variant in the PangoLIN classification [15]. Interestingly, another conserved pattern was also observed in 61/406 sequences in the ORF1ab (R207C, V378I, and M2790I) and classified within the B.4 variant in the PangoLIN classification (Figure 3, green). These CI2 sequences were mainly from the southern part of India (Kerala (*n* = 33), Karnataka (*n* = 6), Leh-Ladakh (*n* = 6), Gujarat (*n* = 2), Maharashtra (*n* = 1), UP (*n* = 1), West Bengal (*n* = 1), and Indian citizens sampled in Iran (*n* = 13). A total of 82.5% of these sequences were observed in March. Guanine (G) was found in 59% of B.4 (CI2 sequences) at position 8 in ORF8, resulting in the G8stop codon mutation in the ORF8 protein along with the ORF1ab (D6270G) amino acid change. These CI2 sequences were mainly identified in Kerala (*n* = 28), Karnataka (*n* = 6), and Gujarat (*n* = 2) during the early period of the pandemic (January–May).

**Figure 4:**
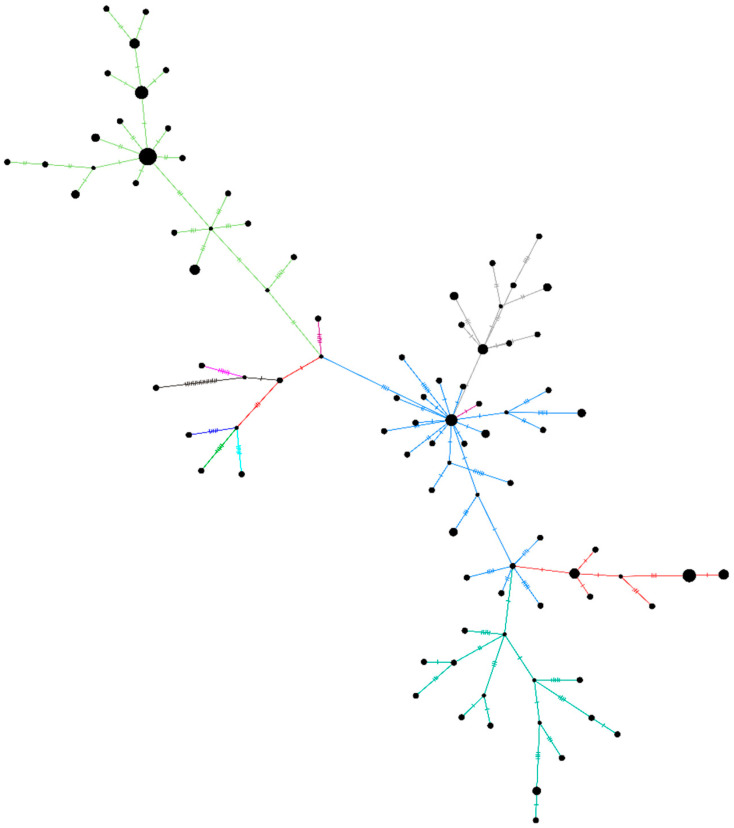
A haplotype network plot was generated from the 96 SARS-Cov-2 sequences belonging to the unclassified cluster, along with representative sequences of the other clades using the median-joining method in PopART v1.7 with epsilon as 0. The light-green color of the branches depicts the B.4 variant of the SARS-CoV-2. Blue-grey, orange, and dark green are the sequences from the B.6 variant.

A haplotype network plot was generated for the unclassified sequences retrieved (*n* = 96) in this study along with the other representative clade sequences (Figure 4). The network plot indicates the presence of two different clusters within the unclassified SARS-CoV-2 sequences. Supplementary Figure S1 depicts the NJ tree for the same set of sequences. The rest of the sequences (*n* = 14) did not have any of the above-described patterns due to the ambiguity in the sequences.

### 3.4. The Temporal Trend of Indian SARS-CoV-2 Sequences Demonstrates an Increase of G and Its Subclades in Different States of India

**Figure 5:**
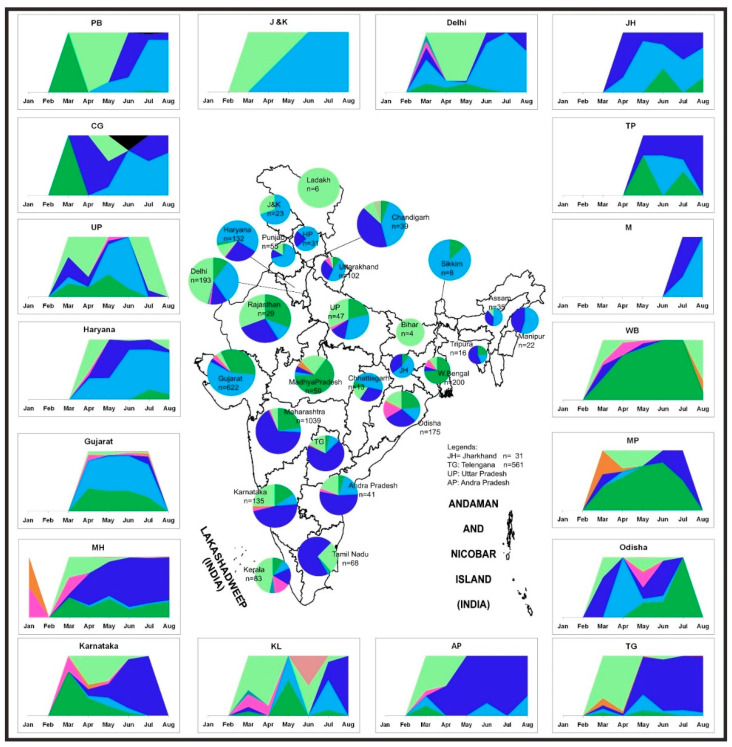
Distribution of the SARS-CoV-2 genome prevalence from the outbreak phase (January 2020) up to the seventh month of the pandemic. Stacked area plots are generated to demonstrate the cumulative temporal trends of the SARS-CoV-2 observed in the different states in India. The x-axis depicts the number of SARS-Cov-2 sequences observed in the respective months. The size of each pie chart within the states of the Indian map is proportional to the numbers in each respective clade. The outline of India’s map is downloaded from http://www.surveyofindia.gov.in/file/Map%20of%20India_1.jpg (accessed on 20 March 2020) and further modified to include relevant data in the SVG editor.

Figure 5 illustrates the temporal trend along with the geographic distribution of the SARS-CoV-2 sequences in India. In the earlier stage of the COVID-19 pandemic, the L, S, and V clade sequences were observed only in Delhi, Maharashtra, and Karnataka states in India. This was followed by the reporting of the G and its subclades from March, an observation similar to previous literature [16,17]. The G clade sequences evolved into two newer subclades (GR and GH). The GR and the GH clades established themselves and superseded their parent strain within only two months (March–May). The predominance of different strains was observed in different states in India. The parental G strain majorly affected Rajasthan, Madhya Pradesh, and Odisha. The southern parts of India (Telangana, Andhra Pradesh, Karnataka, and Maharashtra) seemed to be predominantly affected by the GR strain. The northern states Jammu and Kashmir, Punjab, Chandigarh, Haryana, and Delhi had a prevalence of the GH strains (Figure 5). It was observed that the northern part of India had a higher dominance of the GH clade, whereas the southern and central parts of India had the GR clade [18]. These scenarios were derived from the representative samples, which are fewer in number as compared to the real scenario, hence the limitation to the sample set analyzed.

## 4. Discussion

Whole-genome sequencing (WGS) serves as an important tool for determining geographical prevalence, the evolution of viruses over time, predicting the trends of disease transmission, and for understanding the most effective designs and platforms for developing vaccines and therapeutics [19,20]. WGS also helps in tracing the transmission chains of the virus [21]. The number of cases of COVID-19 is on a continuously rising trend all across the world (1). In India, during the first SARS-CoV-2 wave, a maximum number of cases was reached in the period between September and October 2020, and subsequently declined until February 2021. The recent exponential upsurge (second wave) of the COVID-19 cases in India has been observed from April 2021, with more than 0.2 million new cases being reported as of 17 April 2021 (https://www.worldometers.info/coronavirus/country/india/ (accessed on 22 March 2021)). During the first year of its spread, GISAID classified the virus into different clades based on the specific mutation observed at different protein positions (Figure S2) [22]. However, with the recent diversity of the new SARS-CoV-2 variants, a dynamic nomenclature based on the phylogenetic framework is used to identify lineages with an active spread, referred to as PangoLIN [15].

Three variants as defined in the PangoLIN nomenclature—B.1.1.7 lineage (also known as 20B/501Y.V1 Variant of Concern (VOC) 1 December 2020) and B.1.351 lineage (also known as 20C/501Y.V2) have recently been reported from India [23,24]. These variants are of concern due to antigenic drift, increased transmissibility, and immune escape (especially for B.1.351) mechanisms. The number of variants is increasing, and these strains carry significant mutations in the S gene. We identified a group of strains within lineage B.4, defined by two major changes including a stop codon in ORF8. However, the effect of this unique cluster in disease outcomes or virus transmission has not been ascertained thus far. The analysis of the genome sequences of the SARS-CoV-2 retrieved in this study led to the identification of individual amino acid mutations present in the early samples. Recently, a new PangoLIN lineage (B.1.617) was identified in Indian SARS-CoV-2 sequences, with the E484Q and L452R mutation (commonly known as a double mutant) in the spike protein of SARS-CoV-2, which is considered to have higher transmission rates. The SARS-CoV-2 sequence analyses during the period between January and August 2020 revealed the presence of the E484Q mutation in the spike protein. These sequences were found in Maharashtra in March (*n* = 1) and July (*n* = 2) 2020. Another immune escape mutation, the N440K amino acid in the spike protein, was also observed in Telangana (*n* = 7), Andhra Pradesh (*n* = 5), and Assam (*n* = 1) from May 2020. This indicates that despite the absence of the double mutant variant during the early phase of infection, the presence of a single independent mutation could be seen. Furthermore, it was also observed that the multiple mutations found in the VOC, VUI, and variant of high consequence were not present during this period, although single independent mutations were seen. In addition, this study observed the presence of individual amino acid variants in the SARS-CoV-2 variants B.1.1.7 (S494P), B.1.525 (A67V, Q677H), B.1.526 (L5F, T95I, S477N), and P2 (V1176F) in the earlier samples.

The amino acid mutation—L3606F in the NSP6 region of ORF1ab—is quite intriguing. Most of the strains group in the unclassified cluster. The ORF1ab with L3606 prominently consist of the early S clade sequences, along with the newly emerged G clade and its subclades. Based on the analysis of this study, 84.6% of sequences in the unclassified cluster had the ORF1ab: L3606F mutation. This comprises the B.4 and B.6 lineages. The changes in the amino acid of the remaining SARS-CoV-2 sequences (3.4%) in this set are undefined. The effect at this genomic position needs to be looked upon as limited evolution is observed when Phenylalanine is present at GP 3606 as compared to Leucine. It is observed that the Leucine at the 3606 GP has an increasing number of G and its subclade. This study also identifies the presence of stop codons in the ORF8 protein of the Kerala SARS-CoV-2. The accessory protein ORF8 plays a role in the immune response and evasion, as reported by a recent computational study [25].

This analysis further demonstrates that the SARS-CoV-2 variant was not reported to be circulating in India until August, the samples of which were downloaded on 9th December 2020 [23]. The circulating clades in the country may be attributed to the early introductions into India through travelers as well as the mixing of clades. Besides, the early transmissions within the country could be chiefly traced to movements of migrant workers and the holding of religious gatherings. The independent identification of the amino acid mutations observed in the SARS-CoV-2 variants from the early phase samples indicates an evolutionary trend in the current circulating strain that is geared towards host adaptation. The molecular epidemiology of SARS-CoV-2 needs to be analyzed continuously so that changes in the amino acids can be tracked, and the effect of these mutations in the disease transmission dynamics and its pathophysiology can be promptly assessed.

## Figures and Tables

**Table 1:** Percentage nucleotide and amino acid evolutionary divergence over Sequence Pairs between different GISAID groups, using the P distance along with uniform distribution as the rate variation parameter among sites.

GISAID Clades	Percentage Evolutionary Divergence over Sequence Pairs between Groups									
	L	S	V	Unclassified Cluster	G	GR	GH	GH-GR	GV-GR	GS
**L**		0.081	0.077	0.070	0.077	0.108	0.101	0.112	0.152	0.097
**S**	0.041		0.093	0.088	0.094	0.125	0.117	0.129	0.170	0.114
**V**	0.043	0.050		0.100	0.090	0.122	0.114	0.125	0.166	0.074
**Unclassified Cluster**	0.035	0.045	0.052		0.097	0.128	0.120	0.131	0.173	0.118
**G**	0.043	0.051	0.053	0.051		0.080	0.072	0.085	0.124	0.084
**GR**	0.058	0.066	0.068	0.066	0.047		0.104	0.069	0.100	0.115
**GH**	0.059	0.067	0.069	0.066	0.047	0.063		0.087	0.149	0.105
**GH-GR**	0.060	0.068	0.070	0.068	0.050	0.041	0.055		0.109	0.114
**GV-GR**	0.077	0.085	0.087	0.085	0.066	0.052	0.083	0.056		0.160
**GS**	0.054	0.062	0.040	0.063	0.050	0.065	0.061	0.064	0.085	

## Data Availability

All the sequences are already submitted and are available at the public domain (GISAID database) available from https://www.gisaid.org (accessed on 22 March 2021).

## Supplementary Materials

The following are available online at https://www.mdpi.com/article/10.3390/v13050925/s1 Figure S1 Neighbor-joining of the 96 SARS-CoV-2 sequences belonging to the unclassified cluster using the Tamura-Nei model with a bootstrap replication of 1000 cycles. The light green colour depicts the CI2 (B.4 lineage) variant of the SARS-CoV-2 and Blue and its shades are the sequences from A3i (B.6 lineage) variant. The red colour is a part of the A3i group that has additional missense conservation of S2015R. The representative sequences of L, S, V, G, GH, GR GISAID clades are marked in Red, pink, orange, Green, Blue, and light blue colour respectively. Figure S2. Temporal distribution of the SARS-CoV-2 genome across the world from the outbreak phase (January 2020) till seven (or eight) months of the pandemic. Stacked area plots are generated to demonstrate the cumulative temporal trends of the SARS-CoV-2 observed for different countries of the various continent. The x-axis depicts the number of SARS-Cov-2 sequences observed during respective months. The size of each pie chart within the continent of the world map is proportional to the numbers within each respective clade. The outline of the world map is downloaded from https://d-maps.com/index.php?lang=en and further modified to include relevant data in the SVG editor. Table S1. The patient’s clinical history, for the clinical sample that could retrieve SARS-CoV-2 genomic sequences. Table S2. Details of the patient’s age and gender along with the percentage of the genome retrieved and relevant reads mapped to the SARS-CoV-2 sequence retrieved in this study.
