# Supplementary figures and images for: An Epidemiological Analysis of SARS-CoV-2 Genomic Sequences from Different Regions of India
Source: Viruses. 2021 May 17;13(5):925. doi: 10.3390/v13050925 (PMC8156686; doi:10.3390/v13050925)

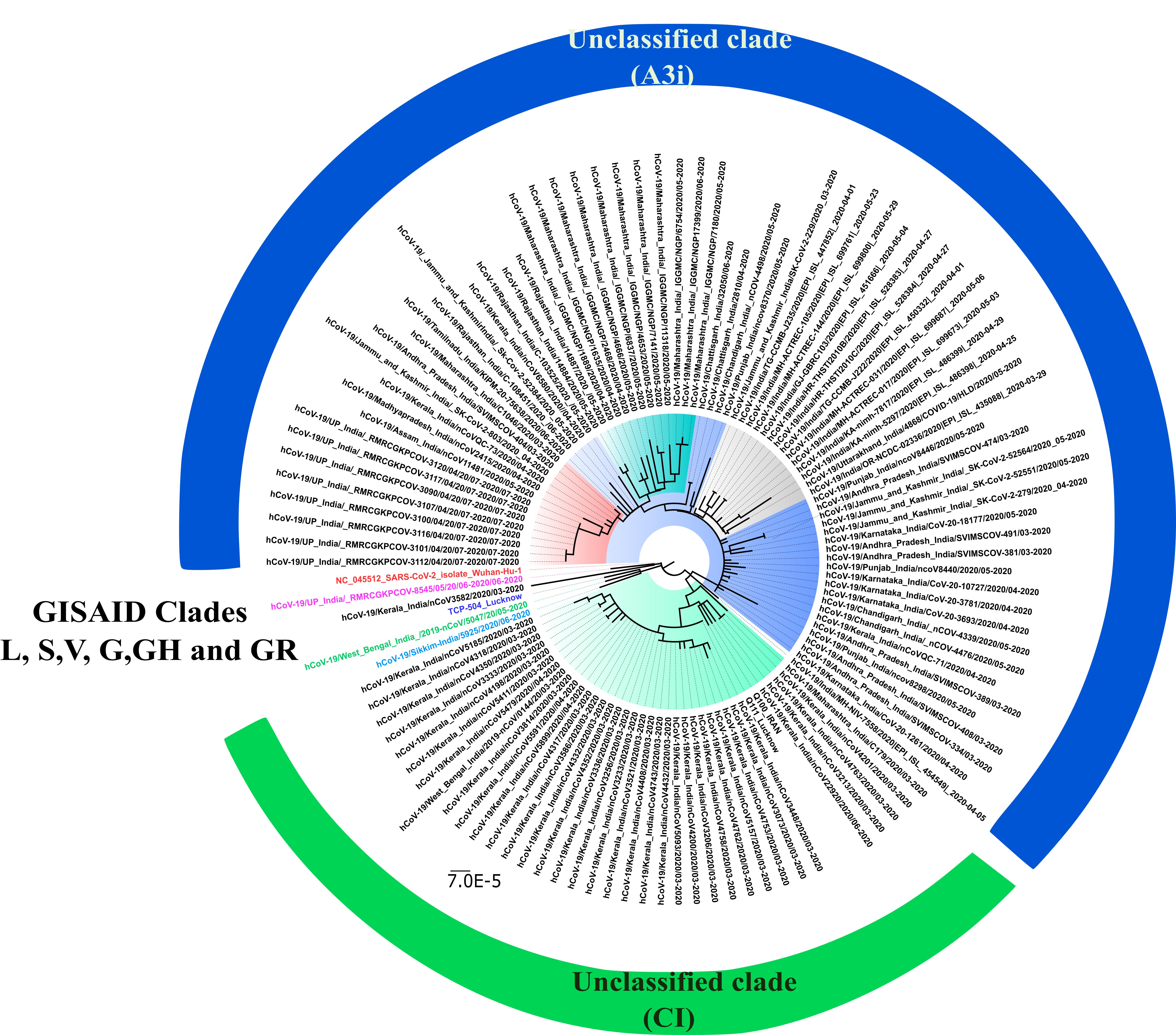

Supplement: Supplementary file 1 [file viruses-13-00925-s001.zip › Supp Fig 1.jpg]

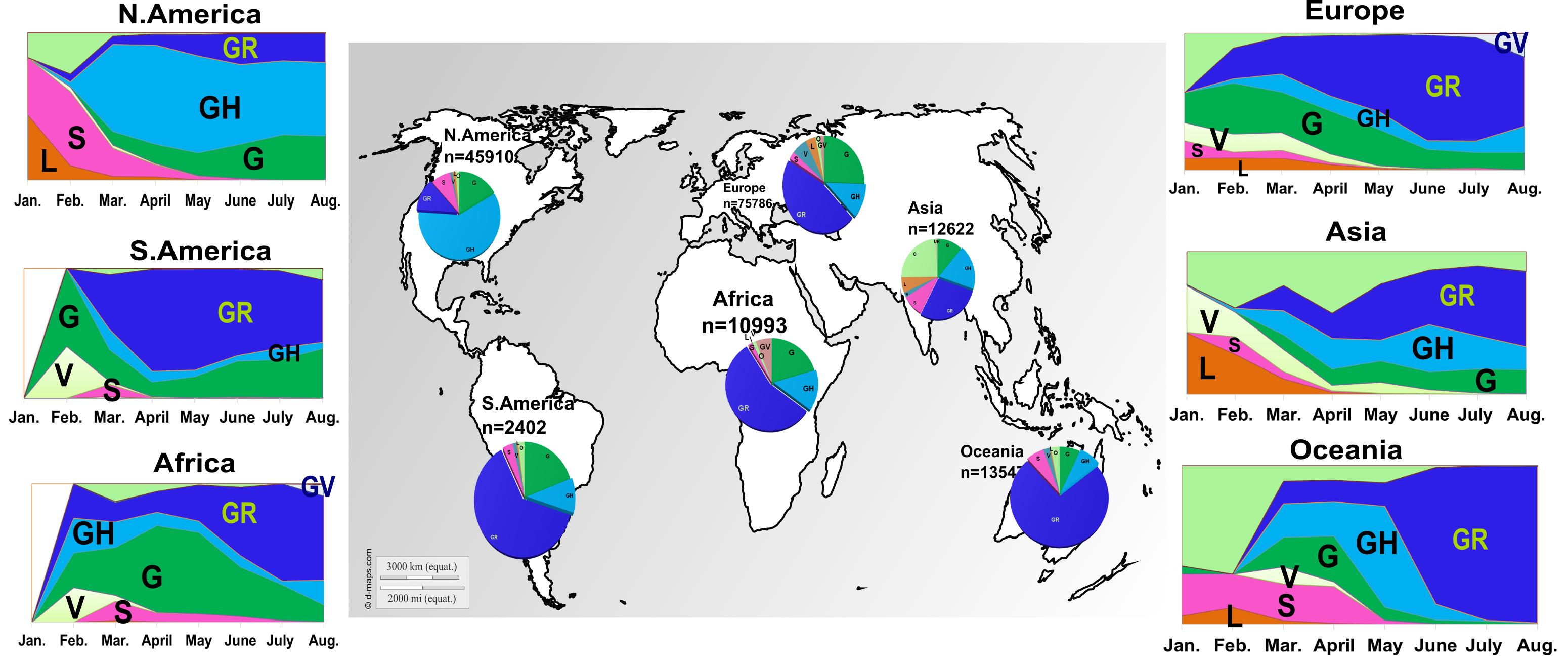

Supplement: Supplementary file 1 [file viruses-13-00925-s001.zip › Supp Fig 2.jpg]
